# How and why non-balanced reciprocity differently influence employees’ compliance behavior: The mediating role of thriving and the moderating roles of perceived cognitive capabilities of artificial intelligence and conscientiousness

**DOI:** 10.3389/fpsyg.2022.1029081

**Published:** 2022-10-20

**Authors:** Nan Zhu, Yuxin Liu, Jianwei Zhang, Jia Liu, Jun Li, Shuai Wang, Habib Gul

**Affiliations:** ^1^School of Business Administration, Fujian Jiangxia University, Fuzhou, China; ^2^Business School, University of International Business and Economics, Beijing, China; ^3^Beijing Institute of Technology, Beijing, China; ^4^Shandong Women’s University, Jinan, China; ^5^Kardan University, Kabul, Afghanistan

**Keywords:** generalized reciprocity, negative reciprocity, thriving at work, perceived cognitive capabilities of artificial intelligence, conscientiousness

## Abstract

Previous studies have paid more attention to the impact of non-balanced reciprocity in the organization on employees’ behaviors and outcomes, and have expected that the reciprocity norm could improve employees’ compliance behavior. However, there are two distinct types of non-balanced reciprocity, and whether generalized reciprocity affects employees’ compliance behavior rather than negative reciprocity and its mechanisms has not been further explored so far. Building on the social exchange theory and cognitive appraisal theory, we established and examined a model in a scenario-based experiment across a two-stage survey of 316 participants. In this article, we propose that generalized reciprocity (relative to negative reciprocity) positively influences employees’ compliance behavior, and thriving at work mediates its relationship. Furthermore, we argue that the positive association between generalized reciprocity and thriving at work is moderated by the perceived cognitive capabilities of artificial intelligence (AI). This association is amplified for people high in the perceived cognitive capabilities of AI. We also propose that the positive association between thriving at work and compliance behavior is moderated by conscientiousness, such that the association is amplified for people high in conscientiousness. These findings have theoretical and practical implications.

## Introduction

Employees’ perceived norm of reciprocity can motivate them to engage in positive work behavior targeted at the initiating actor (e.g., citizenship behaviors; Lavelle et al., 2009; Cropanzano et al., 2017). Furthermore, the organization expects reciprocity norm to improve individuals’ compliance behavior (Lawson and Greene, 2014). Lack of reciprocity can increase employees’ burnout risk and decrease emotional engagement and psychological well-being (van der Ross et al., 2022). Thiel (2021) suggested that the reciprocity norm can be embedded in an organization’s relationship with employees. The social exchange theory (SET) theorists propose that reciprocity can be categorized into two types: *balanced reciprocity and non-balanced reciprocity* (Sahlins, 1972; Gervasi et al., 2022). Constructs that use non-balanced reciprocity are more prevalent than those using balanced reciprocity (Gervasi et al., 2022). This is because, in reality, it is often difficult for the party receiving the help to achieve the immediate and equivalent return demanded by the other party, and the giver cannot tolerate a one-way flow (Sahlins, 1972). Therefore, balanced reciprocity cannot be sustained in the long run. Non-balanced reciprocity has been classified as generalized reciprocity (GR; the organization provides assistance and support without requiring employees’ immediate and equal returns) and negative reciprocity (NR; the organization works against employees’ interests to achieve its aims without punishment) (Sahlins, 1972).

The potential of GR relative to other forms of exchange (e.g., NR) to build strong social relations is the subject of debate in SET (Whitham, 2021). Employees’ compliance with the organization and leaders’ requirements can strengthen social bonds within the organization because this behavior reflects their contributions to the collective pool to form productive exchanges (Whitham, 2021). However, whether GR relative to NR influences employees’ compliance behavior has not yet been investigated. Employees’ compliance behavior can improve the effectiveness of management policies and operational efficiency (D’Arcy and Lowry, 2019). This study investigates the effect of GR (relative to NR) on employees’ compliance behavior and its mechanism by considering their different exchange values. Exploring these issues could help organizations improve employees’ compliance behavior from an exchange norm perspective.

Previous literature suggests that there are two different approaches to fostering employee rule adherence: the extrinsic command-and-control approach and intrinsic self-regulatory approach (Tyler and Blader, 2005). Most studies have explored the antecedents of employees’ compliance behavior from an extrinsic command-and-control perspective, such as response cost, sanction severity (Chen et al., 2018), instrumental culture (Spitzmuelle and Stanton, 2006), and reward and punishment expectations (Liang et al., 2013; Li et al., 2021). However, few studies have investigated its antecedents from an intrinsic self-regulatory viewpoint, such as job autonomy (Li et al., 2021), perceived usefulness (Xue et al., 2011), and management participation (Hwang et al., 2021). An intrinsic self-regulatory approach may enable employees to comply voluntarily with the rules. Reciprocity was labeled as “intrinsic” and “instrumental (or extrinsic)” (Sobel, 2005; Segal and Sobel, 2007; Cabral et al., 2014). For intrinsic reciprocity, a kind (unkind) act by one agent changes the preferences of the people they interact with in such a way as to elicit kindness (unkindness) (Cabral et al., 2014). GR and NR are intrinsic because they reflect organizations’ altruism and self-interest, respectively (Sahlins, 1972). Previous studies showed that they could differently influence employees’ psychological states and behaviors, such as autonomous or intrinsic motivation (Liu et al., 2021), deviant (Restubog et al., 2010), or cheating behavior (Kamran et al., 2022), affective commitment (Quratulain et al., 2018), and social entrepreneurship intention (Xiang and Zhang, 2022). Previous studies have demonstrated a generic model of SET social exchange (Cropanzano et al., 2017). This model indicates that if an organization provides benefits to employees, employees tend to provide benefits to the organization in return; if the organization harms employees, they also do harm to the organization (Cropanzano et al., 2017). However, comparing the effect of organizations’ actions on employees’ beneficial behavior has been ignored. For example, how and when does GR (relative to NR) influence employees’ compliance behavior? The two types of non-balanced reciprocity, GR and NR are both widely embedded in the organization, which uses them to guide employees’ compliance behaviors. Exploring the effect of GR (relative to NR) could help us extend the generic model of SET social exchange (Cropanzano et al., 2017). In particular, compared to the negative actions of an organization toward employees, the positive actions of an organization toward employees are more likely to affect employees’ beneficial behavior.

However, how and why GR (relative to NR) influences employees’ compliance behavior has not yet been explored. Employees’ compliance behavior has been found to influence business unit performance (Wu et al., 2014). Exploring this issue could help practitioners guide employees’ adherence to rules and policies through an intrinsic self-regulatory approach. This study is also beneficial for researchers to better understand the non-balanced reciprocity from an intrinsic viewpoint.

**Figure 1:**
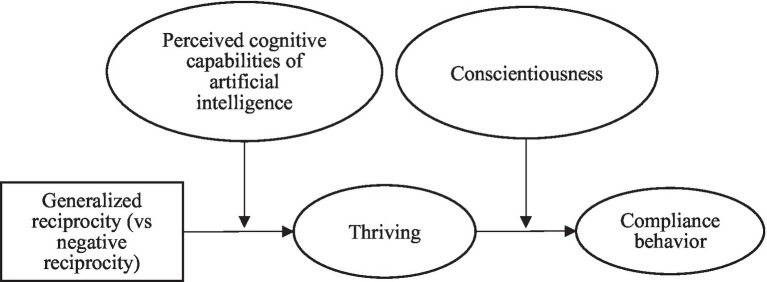
The theoretical model.

In this study, based on SET (Blau, 1964) and cognitive appraisal theory (CAT) (Lazarus and Folkman, 1984; Chiu et al., 2021), we establish a theoretical model (Figure 1) that hypothesizes that GR (relative to NR) may create employees’ productive exchange or benefits to the organization and thus positively influence their compliance behavior. We also hypothesized that GR (relative to NR) could promote employees’ self-regulatory psychological mechanism, especially the self-regulatory psychological state. Thriving at work (i.e., individuals’ learning and vitality experiences) reflects that individuals can autonomously self-regulate their feelings (Spreitzer et al., 2005). SET literature suggests that thriving can mediate the relationship between exchange relations (e.g., leader-member exchange) and employee behavior (e.g., workplace deviance; Walumbwa et al., 2020). GR (relative to NR) may make them perceive development and vigor (i.e., thriving), which in turn influences compliance behavior. Furthermore, the SET literature suggests that technology can influence individuals’ social exchange process (Mitchell et al., 2012), while it also causes psychological stress to employees. As an advanced technology, artificial intelligence (AI) is valued by organizations because of its capability, and its use may make employees fear they may be replaced. Therefore, employees tend to appraise AI’s cognitive capabilities. The CAT proposes that individuals can exert cognitive appraisal of stressful situations to influence their psychological state (Lazarus and Folkman, 1984; Chiu et al., 2021). Therefore, by integrating SET with CAT, we hypothesized that the perceived cognitive capabilities of AI amplified the effect of GR on thriving at work. Employees may conduct a cognitive evaluation on AI, which in turn intervenes in the relationship between GR and thriving. Furthermore, the thriving literature on SET demonstrated that personality can significantly moderate the thriving–behavior relationship (Zhang et al., 2018). Conscientiousness has been shown to moderate the link between psychological state and outcomes (Lin et al., 2015). Therefore, we argue that conscientiousness may amplify the effect of thriving on employees’ compliance behavior.

Our research has the following theoretical implications. First, our research expands the antecedents of compliance behavior using an intrinsic self-regulatory approach (Tyler and Blader, 2005; Hwang et al., 2021; Li et al., 2021). Second, the findings indicate that non-balanced reciprocity norms labeled as intrinsic have a wider scope of implications than those studied so far (e.g., affective commitment, turnover intention). Third, this study confirms the mediating role of thriving in the relationship between GR and compliance behavior, thus expanding the literature on thriving in SET (Walumbwa et al., 2020). Fourth, by integrating SET (Blau, 1964) and CAT (Lazarus and Folkman, 1984; Chiu et al., 2021), our study demonstrates the role of employees’ differences in their cognitive appraisal of challenging technology and personality trait, namely, the perceived cognitive capabilities of AI and conscientiousness, which tend to condition some of the self-regulatory psychological state and behavior outcomes of non-balanced reciprocity norms.

## Theoretical development and hypotheses

### The effect of generalized reciprocity (relative to negative reciprocity) on employees’ compliance behavior

Based on SET, a generic model of social exchange proposes two paths of exchange: if an organization provides benefits to employees, employees tend to engage in positive behaviors or outcomes for the organization; and if an organization harms employees, they may show negative behaviors or results for the organization (Cropanzano et al., 2017). Previous literature suggests that GR and NR differently influence employees’ behaviors (Restubog et al., 2010; Kamran et al., 2022). However, the potential of GR relative to other forms of exchange (e.g., NR) to build strong social relations is the subject of debate in SET (Whitham, 2021). Based on SET, we hypothesized that GR (relative to NR) has a positive effect on employees’ compliance behavior. Compared to NR, which is characterized by self-interest, GR takes on the characteristic of giving without requiring a return (Sahlins, 1972), making employees perceive organizational altruism. The organization implementing NR does not have to worry about being punished by employees (Sahlins, 1972) so that employees will not feel that they are interdependent with the organization. Employees under GR (relative to NR) tend to increase intrinsic self-regulation because they perceive their interdependence with the organization which generates positive sentiments (e.g., trust, affective regard) to increase integrative social bonds (Whitham, 2021). For example, previous literature has shown that GR can significantly increase individuals’ perception of giving from organization, which in turn improves the perception of interdependence (Whitham, 2021). Consequently, employees are likely to increase their intentions to maintain their connection with the organization. Thus, employees under GR (relative to NR) are willing to comply with the rules and policies of the organization and leaders as a response. Empirical research has suggested that NR can significantly result in non-compliance behavior (e.g., cyberloafing) (Koay et al., 2022). Therefore, we hypothesize as follows that:

*H1*: Generalized reciprocity (relative to negative reciprocity) has a positive effect on employees’ compliance behavior.

### The effect of generalized reciprocity (relative to negative reciprocity) on thriving and the mediating role of thriving

To further explore whether GR has more potential than another exchange norm (e.g., NR) in establishing strong social relations (Whitham, 2021) and its psychological mechanism, we propose that GR (relative to NR) can positively influence employees thriving at work, and thriving tends to mediate the relationship between GR and compliance behavior. The literature based on SET suggests that GR may activate a bond-building or productively psychological mechanism (Whitham, 2021). Thriving can be seen as a productive psychological state because employees who are thriving perceive they are learning new knowledge and feel vigor at work (Spreitzer et al., 2005). Based on SET, we propose that GR (relative to NR) can increase employees’ thriving because of the autonomous self-regulation. A previous study has demonstrated that GR (relative to NR) could increase employees perceived organizational support (Liu et al., 2021) which can prompt employees’ autonomous self-regulation (i.e., autonomous motivation) (Gagné and Deci, 2005). Autonomous motivation is a self-regulatory psychological process (i.e., people work out of intrinsic interest and identify with organizational culture) (Deci and Ryan, 2000) that helps employees perceive positive emotion which in turn encourages employees to learn new knowledge and feel energetic (i.e., thriving at work) (Mukhaimer, 2012). Thus, GR (relative to NR) can increase employees’ thriving at work.

Second, based on SET, the current literature suggests that thriving tends to mediate the relationship between social exchange or situation and employee behavior (Chang and Busser, 2020; Walumbwa et al., 2020). We argue that thriving may mediate the relationship between GR and compliance behavior. Thriving at work can significantly influence employees’ career adaptability (Jiang, 2017). This is because thriving employees can learn knowledge and feel energy during the work, which may prompt them to comply with organizational and leaders’ rules in return for the organization.

*H2a*: Generalized reciprocity (relative to negative reciprocity) has a positive effect on employees’ thriving at work.

*H2b*: Thriving at work mediates the beneficial effect of generalized reciprocity on employees’ compliance behavior.

### The moderating effect of perceived cognitive capabilities of artificial intelligence in the relationship between GR and thriving at work

The literature on SET suggests that technology influences the psychological processes of social exchange (Mitchell et al., 2012). The current study also suggests that technological innovation plays a role in the effect of reciprocity norms within the organization (Yu et al., 2022). AI as an advanced technology is highly valued by organizational practitioners, while the actual or potential use of AI in the organization will make employees feel the pressure of being replaced. The CAT posits that individuals can make cognitive evaluations of a challenging or stressful situation and then influence their psychological states (Lazarus and Folkman, 1984; Chiu et al., 2021). Individuals also have different levels of cognitive evaluation of AI (Chiu et al., 2021). Specifically, the perceived cognitive capabilities of AI refer to the fact that employees realize that AI understands the organizational context and human language and also provides transparency into how the decisions and recommendations are made (Chiu et al., 2021). Therefore, by integrating SET (Blau, 1964) and CAT (Lazarus and Folkman, 1984; Chiu et al., 2021), we propose that the perceived cognitive capabilities of AI tend to strengthen the effect of GR on thriving because of perceived organizational support. Specifically, in the GR situation, employees who perceive AI to have high cognitive capabilities increasingly perceive organizational support, prompting them to thrive at work. The perceived cognitive capabilities of AI can significantly increase the cognitive attitude of AI (e.g., AI is a wise and beneficial technology) (Chiu et al., 2021), which may help employees working in GR organizations perceive the organization’s altruism and support. This is because employees who score high in the perception of the cognitive capabilities of AI believe that organizations investigate technology to improve their work efficacy. Thus, the perceived cognitive capabilities of AI enable employees working in GR to perceive an increasing trend of organizational support and then perceive a higher level of thriving at work (Chang and Busser, 2020). The empirical literature on social exchange shows that organizational support is significantly associated with employees’ thriving at work (Chang and Busser, 2020).

*H3*: The positive association between generalized reciprocity and thriving at work is moderated by the perceived cognitive capabilities of AI such that this association is amplified for people high in the perceived cognitive capabilities of AI.

### The moderating effect of conscientiousness in the relationship between thriving at work and compliance behavior

The empirical literature on SET demonstrates that personality can significantly moderate the effect of thriving on employees’ behavior and outcomes (Zhang et al., 2018). Conscientiousness has been shown to moderate the association between an individual’s attitudes, behaviors, and outcomes (Tziner et al., 2002; Lin et al., 2015). Based on SET (Blau, 1964), we propose that conscientiousness can amplify the effect of thriving at work on employees’ compliance behavior because of career adaptability. Individuals high in conscientiousness are likely to regulate their behavior due to a sense of responsibility, which increases moral reflectiveness (Kim et al., 2014) to influence leader-member exchange. Empirical research has demonstrated that individuals high in conscientiousness can perceive a high level of leader-member exchange (Kamdar and Van Dyne, 2007) to increase career adaptability (Yang et al., 2019). Thus, employees with a high level of conscientiousness, combined with thriving at work, show increased career adaptability. Such employees are more willing to be productive and comply with organizational policies and leaders’ orders.

*H4*: The positive association between thriving at work and compliance behavior is moderated by conscientiousness such that the association is amplified for people high in conscientiousness.

## Methods

### Participants and procedure

This study was conducted at two different time points using a scenario-based experimental method. Participants were undergraduates of universities in China. Before the study, the researchers introduced the main purpose and content of the study to the participants and stated that the study would be conducted at two different times. The researchers explained that the experiment-based surveys were only for academic research and were confidential, and participation was voluntary and anonymous. Participants could withdraw at any time during the investigation. At Time 1, the researchers required the participants not to provide any of their personal information on identity. After that, the researchers explained that to ensure the combination of questionnaires and protect their privacy, participants could provide the year and month of birth of someone they considered important and hoped that they could remember this information seriously and ensure this confidentiality. The researchers advised them not to reveal who the person referred to in the survey was or talk about it with others to guarantee confidentiality, anonymity, and uniqueness. Researchers explained that the information would be used during the scenario-based experiment at Time 2. At Time 2, the researchers invited participants who finished the survey at Time 1 to attend the scenario-based experiment and also provided the date used in the Time 1 survey.

Participants were invited to provide their demographic information and moderators, and another control variable during the survey at Time 1 (i.e., age, gender, perceived cognitive capabilities of AI, conscientiousness, extroversion). They were also informed that if they participated at Time 1, they would be invited to attend the scenario-based experiment at Time 2. At Time 2, participants who completed the survey at Time 1 were divided into two groups and invited to read the corresponding scenarios. Participants were invited to imagine that they worked in the corresponding scenario (i.e., one reflected the GR norm within the organization, and the other reflected the NR norm within the organization). Subsequently, they were invited to answer the independent variable (i.e., GR, NR), mediator (i.e., thriving at work) and then answered the dependent variable (i.e., compliance behavior). 372 participants were invited to take part in this research and finally, 316 participants finally attended the research (effective response rate = 84.9%) (49.1% were male, and the mean age was 20.8).

### Measures

We invited participants to answer all the items on the variables in our research using a seven point 7-Likert scale (from 7 = strongly agree to 1 = strongly disagree).

### Generalized reciprocity and negative reciprocity

We measured GR using a four-item scale and NR using the seven-items scale developed by Wu et al. (2006). Sample items are “My organization would do something for me without any strings attached” and “My organization would never help me out unless it was in the organization’s own interest.” Cronbach’s alpha was 0.94, 0.92, respectively.

### Thriving at work

We measured thriving at work using a ten-item scale developed by Porath et al. (2012), which included five items for the learning dimension and five items for the vitality dimension, respectively (e.g., “I continue to learn more as time goes by”). Cronbach’s alpha = 0.93.

### Perceived cognitive capabilities of artificial intelligence

We measured the perceived cognitive capabilities of AI using the nine-items scale developed by (Chiu et al., 2021). It was composed of three items each for natural language processing, understanding context, and logic transparency, respectively (e.g., “I think the AI system could process languages and texts like a human”). Cronbach’s alpha was 0.86.

### Conscientiousness

We measured conscientiousness using a four-item scale developed by Donnellan et al. (2006) (e.g., “I get chores done right away”). Cronbach’s alpha was 0.82.

### Compliance behavior

We measured compliance behavior using a three-item scale developed by Murphy and Tyler (2008). An example of a reverse item is “neglect to follow work rules or the instructions of your supervisor.” Cronbach’s alpha = 0.81.

### Control variables

We selected the age and gender of the participants as control variables. Previous studies had demonstrated that individuals high in extraversion could perceive an increasing trend of thriving at work than low (Hennekam, 2016). Therefore, when we examined the direct effect of GR on thriving (H2a) and the moderating effect of perceived cognitive capabilities of AI in the relationship between GR and thriving (H3), we controlled for extraversion (e.g., “I am the life of the party,” Cronbach’s alpha = 0.81; Donnellan et al., 2006).

### Construct validity

Following Sheng et al. (2018) approach, we estimated the average variance extracted values (AVE) (Fornell and Larcker, 1981) and composite reliability (CR) (Bagozzi and Yi, 1988) of all latent constructs, and their values exceeded the thresholds (AVE > 0.5, CR > 0.7), thus supporting the convergent validity and reliability. Each square of the correlations of all constructs was less than their AVE, thereby supporting the discriminant validity of all latent variables (Fornell and Larcker, 1981). Furthermore, we compared the five-factor model with the one-factor model by conducting confirmatory factor analysis (CFA) in Mplus 7.0 (Table 1). The results showed that the five-factor model had a better fit than the one-factor model (χ^2^ = 729.76, df = 391, TLI = 0.92, SRMR = 0.06, CFI = 0.93, RMSEA = 0.05), further supporting the discriminant validity of the latent variables.

**Table 1:** The results of fit indices of confirmative factor analysis of measurements.

Model	χ^2^	d*f*	∆χ^2^	∆d*f*	SRMR	TLI	CFI	RMSEA
Five-factor model	729.76	391			0.06	0.92	0.93	0.05
Four-factor model	1118.17	395	388.41	4	0.08	0.83	0.85	0.08
Three-factor model	1397.48	399	667.72	8	0.14	0.77	0.79	0.09
Two-factor model	1715.44	401	985.68	10	0.15	0.70	0.73	0.10
One-factor models	3125.50	405	2395.74	14	0.15	0.39	0.43	0.15

** *p* < 0.01.

In five-factor model, each latent construct was loaded on each factor. In four-factor model: conscientiousness and extraversion were merged into one factor. In three-factor model and based on four-factor model, perceived cognitive capabilities of AI and thriving were merged into one factor. In two-factor model and based on three-factor model, conscientiousness, extraversion, and compliance behavior were merged into one factor. In one-factor model, all latent constructs were merged into factor.

### Common method bias and manipulation check

We addressed the concerns of common method bias by adopting the psychological and temporal separations of the measurements. First, following Podsakoff et al. (2003) method, we conducted an experimental scenario-based survey by manipulating two different cover stories to create a psychological separation, thus making the measurements of predictor variables independent of or uncorrelated with the measurements of other criterion variables. Second, following Podsakoff et al. (2003) approach, we conducted the surveys at two different times, thus making the measurements of moderator variables and demographics temporally separate from those of the other criterion variables. Third, the model fit results of the five-factor model were better than those of the one-factor model (Table 1), thereby addressing the serious impact of common method bias in our study.

The results of the *t*-test showed that participants in the GR condition (SD = 1.11, mean = 4.86) scored a higher value of GR than those in the NR condition (SD = 1.02, mean = 2.41, *t* (311) = 20.37, *p* < 0.01). Furthermore, participants in the NR condition (SD =1.07, mean = 5.04) scored a higher value of NR than those in the GR condition (SD = 0.94, mean = 3.38, *t* (310) = −14.62, *p* < 0.01), thus supporting the effectiveness of our manipulation.

## Analyses and results

Correlations, standard deviations, and mean values were estimated by using SPSS 22.0 and shown in Table 2. These results were consistent with our hypotheses (H1, 2a, and 2b).

**Table 2:** Standard deviations, means, and correlations among the variables.

Variables	Mean	*SD*	1	2	3	4	5	6	7
Age (T1)	20.81	1.34							
Gender (T1)	1.51	0.50	0.02						
Extraversion (T1)	3.94	0.97	−0.06	−0.11					
Non-balanced reciprocity (T2) (GR = 1, NR = 0)	0.50	0.50	−0.02	−0.06	0.01				
Thriving at work (T2)	4.39	0.95	−0.02	−0.07	0.02	0.49**			
Perceived cognitive capabilities of AI (T1)	4.54	0.78	−0.03	−0.01	0.10	0.04	0.17**		
Conscientiousness (T1)	4.30	0.95	−0.01	0.04	0.12*	0.00	0.12*	0.10	
Compliance behavior (T2)	4.42	1.47	−0.01	−0.02	0.04	0.47**	0.38**	−0.02	0.19**

* *p* < 0.05;

** *p* < 0.01.

T represents the time of the experiment-based questionnaire survey. GR, generalized reciprocity; NR, negative reciprocity.

Following Sheng et al. (2018) and Palmatier et al. (2007) approach, we examined our theoretical model (Figure 1) by conducting maximum likelihood estimation through structural equation modeling (SEM) in Mplus 7.0. The reasons are as follows: first, we can test our hypotheses by simultaneously using all latent variables in an entire model rather than separately; second, we can use latent variables in SEM to explain measurement errors rather than residual error terms by aggregating measurement errors (Sheng et al., 2018).

The results of the SEM in Model 1 showed that GR was positively related to compliance behavior (*B* = 0.543, *p* < 0.01; χ^2^ = 16.61, df = 6, TLI = 0.95, SRMR = 0.03, CFI = 0.98, RMSEA = 0.07) (Table 3). Accordingly, H1 was supported. Model 2 showed that GR was also positively related to thriving at work (*B* = 0.539, *p* < 0.01; χ^2^ = 256.48, df = 114, TLI = 0.94, SRMR = 0.05, CFI = 0.95, RMSEA = 0.06). So, H2a was supported. Furthermore, in Model 3, we examined whether GR has an indirect effect on compliance behavior through thriving at work using a bootstrapping test in Mplus 7.0 (Preacher and Hayes, 2008; Zhao et al., 2010). The results showed that GR was positively related to thriving at work (*B* = 0.539, *p* < 0.01) and compliance behavior (*B* = 0.408, *p* < 0.01). The 95% bias-corrected confidence interval showed that GR has an impact effect on compliance behavior [0.788, 1.534] through thriving at work [0.833, 1.285] (*N* = 5,000), thus supporting H2b.

**Table 3:** The direct and mediating effect analyses.

Independent variable	Model 1		Model 2		Model 3			
	Direct effect				Mediating effect			
	Compliance behavior (T2)		Thriving at work (T2)		Thriving at work (T2)		Compliance behavior (T2)	
	*B*	SE	*B*	SE	*B*	SE	*B*	SE
Age (T1)	0.009	0.056	−0.019	0.039	−0.019	0.037	0.014	0.056
Gender (T1)	0.017	0.151	−0.041	0.106	−0.043	0.108	0.028	0.154
Extraversion (T1)			0.017	0.075	0.019	0.088		
Non-balanced reciprocity (T2) (GR = 1, NR = 0)	0.543**	0.155	0.539**	0.108	0.539**	0.115	0.408**	0.190
Thriving (T2)							0.254**	0.117
χ2	16.61		256.48		321.64			
d*f*	6		114		161			
SRMR	0.03		0.05		0.06			
TLI	0.95		0.94		0.94			
CFI	0.98		0.95		0.95			
RMSEA	0.07		0.06		0.06			

** *p* < 0.01.

The standardized estimates and residuals were shown in this table. GR, generalized reciprocity; NR, negative reciprocity.

Following Sheng et al. (2018) and Palmatier et al. (2007), we examined the moderating roles of the perceived cognitive capabilities and conscientiousness by using multigroup structural analyses. First, the median value of perceived cognitive capabilities of AI was 4.56, which was used to divide all samples into high and low groups. Participants with low or high perceived cognitive capabilities of AI were assigned to low (*n* = 143) and high (*n* = 156) groups, respectively. We examined their moderating effects by comparing the unconstrained model with the constraint model using chi-square difference. Specifically, the coefficient related to the association of concern was constrained to keep equal in the two groups vs. an unconstrained model in which the coefficients were estimated freely in the two groups. Table 4 shows that a high level of perceived cognitive capabilities of AI amplified the effect of GR on thriving at work (*B* = 0.644, *p* < 0.01) than low (*B* = 0.441, *p* < 0.01, △χ^2^/△*f* = 5.408, *p* < 0.05), thus supporting H3a. Second, the median value for conscientiousness was 4.25. Participants with low or high conscientiousness were assigned to low (*n* = 122) and high (*n* = 145) groups. Table 4 shows that a high level of conscientiousness strengthened the influence of thriving at work on compliance behavior (*B* = 0.276, *p* < 0.05) more than a low level (*B* = 0.736, *p* < 0.01, ∆χ^2^/∆*f* = 4.983, *p* < 0.05), so supporting H3b.

**Table 4:** The moderating effects analyses.

Perceived cognitive capabilities of AT	B (low group)	B (high group)	∆χ^2^(∆df = 1)
GR → Thriving at work	0.441**	0.644**	5.408*
Conscientiousness			
Thriving at work → Compliance	0.276*	0.736**	4.983*

* *p* < 0.05;

** *p* < 0.01.

Path estimates are standardized. Parentheses show standard errors; ∆χ^2^ shows that the difference of χ^2^ between the unconstrained model and constrained model.

## Discussion

The findings confirmed the effects of GR (relative to NR) on employees’ compliance behavior and thriving at work. The results also confirmed the mediating role of thriving at work in the positive association between GR and compliance behavior. In addition, employees high in the perceived cognitive capabilities of AI amplify the positive effect of GR on thriving at work. Furthermore, employees high in conscientiousness also strengthen the positive relationship between thriving at work and compliance behavior.

### Theoretical contributions

First, our research expands the antecedents of compliance behavior from an intrinsic self-regulatory approach (Tyler and Blader, 2005; Hwang et al., 2021; Li et al., 2021), expanding the literature on compliance behavior in SET (Blau, 1964). Most prior studies explored the antecedents of compliance behavior from an extrinsic command-and-control approach, such as reward and punishment expectations (Liang et al., 2013; Li et al., 2021) and sanction severity (Chen et al., 2018), and instrumental culture (Spitzmuelle and Stanton, 2006). Few studies have investigated its antecedents from an intrinsic self-regulatory viewpoint, such as job autonomy (Li et al., 2021) and management participation (Hwang et al., 2021). SET posits that the organization offers benefits to individuals, and individuals return the benefits to the organization (Cropanzano et al., 2017). Our research examined GR (relative to NR) as the relationship norm trigger for compliance behavior. Our findings provide significant support for the proposal that the non-balanced norm can help employees regulate themselves to improve their compliance behavior. This is because, under the GR (relative to NR) norm, employees can perceive interdependence with the organization, which influences their perception of social bonds to comply with rules and policies. Accordingly, our research expands the literature on the compliance behavior of SET.

Second, the findings indicate that non-balanced reciprocity norms labeled as intrinsic have wider implications than those studied thus far (e.g., affective commitment and turnover intention). Non-balanced reciprocity can be seen as intrinsic reciprocity because GR and NR reflect organizations’ altruism or self-interest, which in turn elicits employees’ corresponding behaviors and outcomes (Cabral et al., 2014). The majority of the literature on non-balanced reciprocity focused on the individuals’ outcomes and psychological states (e.g., performance and turnover intention; Zhao, 2010; Quratulain et al., 2018), while few studies have focused on its effect on individuals’ behavior, such as deviant behavior (Restubog et al., 2010). Our findings suggest that GR (relative to NR) has an impact on individuals’ self-regulatory behavior during work (i.e., compliance behavior). Our research can enhance understanding of non-balanced reciprocity from an intrinsic perspective, thus expanding the literature on non-balanced reciprocity in SET (Sahlins, 1972; Gervasi et al., 2022). Our findings also align with generosity research (Whitham, 2021). Furthermore, this research can help us extend the generic social exchange model of SET (Cropanzano et al., 2017). Our findings provide empirical evidence to support that compared to the negative action of an organization toward employees, the positive action of an organization toward employees is more likely to affect employees’ beneficial behavior.

Third, this study confirms the mediating role of thriving in the relationship between GR and compliance behavior, thus expanding the literature on thriving in SET (Walumbwa et al., 2020). Existing studies have demonstrated that individuals’ thriving at work, as an internal self-regulatory psychological state (Porath et al., 2012), could be the mechanism linking exchange relationship perception and outcomes at work (Xu et al., 2017; Chang and Busser, 2020). This finding is theoretically important because exchange relationship perception can affect an individuals’ psychological state of learning and vitality and then elicit outcomes as a reward for this relationship. However, most studies on the exchange mechanisms focus on affective commitment and emotions as the mechanisms linking reciprocity to individuals’ behavior and outcomes (Meier and Semmer, 2013; Quratulain et al., 2018; Whitham, 2021). Our findings suggests that the self-regulatory psychological consequences of how non-balanced reciprocity may have wide behavioral implications. Accordingly, our research expands the literature on thriving in SET (Walumbwa et al., 2020).

Fourth, by integrating SET (Blau, 1964) and CAT (Lazarus and Folkman, 1984; Chiu et al., 2021), our study demonstrates the role of employees’ differences in the evaluation of technology and personality trait, namely, perceived cognitive capabilities of AI and conscientiousness, which tend to moderate some of the self-regulatory psychological states and behavior outcomes of non-balanced reciprocity norms. Previous studies have primarily focused on societal identification and risk perception which can intervene in the impact of reciprocity on employees’ psychological states (Neumann, 2019; Whitham, 2021). The literature on SET suggests that technology influences the process of exchange (Mitchell et al., 2012). Our findings highlight that individuals’ cognitive appraisal of AI (i.e., perceived cognitive capabilities of AI) can amplify the influence of non-balanced reciprocity on individuals’ thriving at work. This is theoretically important because individuals’ appraisal of technology can reflect their evaluation of the organization’s investment relationship, which can intervene in the effect of non-balanced reciprocity on their psychological state. Furthermore, consistent with the personality traits of SET (Zhang et al., 2018), our study also confirms that personality traits (i.e., conscientiousness) can moderate the relationship between thriving and compliance behavior, thus further indicating that personality can interfere with the thriving effect.

### Practical implications

Our research provides empirical insights for practitioners regarding how organizations guide employees to regulate their psychological state and influence compliance behavior from a relationship norm perspective. First, the results demonstrate that the GR as an effective relationship norm can be an intrinsic self-regulatory approach to increase employees’ compliance behavior. In other words, employees under GR can perceive organizational altruism to increase interdependence with the organization and autonomously achieve their rules or policy adherence.

Second, our findings show that thriving at work can link the relationship between GR and compliance behavior. This means that organizations can pay attention to employees’ psychological changes in learning and vitality which reflect employees’ internal self-regulation under GR and then influence compliance behavior. Previous studies on SET have demonstrated that leader-member exchange can influence employees’ workplace deviance by facilitating employees’ thriving at work (Walumbwa et al., 2020). This research further highlight the significance of establishing exchange relation norms for employees’ internal self-regulatory states.

Third, the results of this study suggest that the perceived cognitive capabilities of AI and conscientiousness can facilitate the increasing trends of employees’ thriving at work and compliance behavior under GR. The organization can invest in AI, and enhance employees’ thriving under GR by improving their recognition of the cognitive ability of AI. Furthermore, our research also found that when employees are thriving at work, those with high conscientiousness tend to increase their level of compliance with rules and policies. Accordingly, the organization should consider the importance of conscientiousness in forming individuals’ work behavior and advocate practitioners to understand the interference of conscientiousness in their work behavior.

### Limitations and future research

Our study has several limitations; therefore, we also provide the future research directions. First, our research conducts a laboratory-based experimental design. This design has many advantages for the research on non-balanced reciprocity: it allows researchers to effectively manipulate the intended construct (i.e., GR vs. NR) and facilitate the estimation of its causal effect (Bamberger and Belogolovsky, 2017). However, the lab-based experimental design limits the external validity of the findings (Bamberger and Belogolovsky, 2017). Future research could consider achieving external validity by conducting a large-scale questionnaire survey in different enterprises and countries.

Second, we measured the variables by collecting self-reported data from a single source. Due to the limited data sources and self-report measurements, our research has the possibility of common method bias (Podsakoff et al., 2003). However, we controlled for its serious influence through the psychological and temporal separations of the measurements as suggested by Podsakoff et al. (2003). The CFA results further demonstrate that our research does not have a serious common method bias (Table 1). Future studies should consider the multiple data sources and objective measurements.

Third, based on SET (Blau, 1964), researchers can investigate other possible mediating and moderating mechanisms, such as trust (Wang et al., 2022). Specifically, trust can significantly mediate the relationship between fairness perceptions and outcomes (Wang et al., 2022). GR (relative to NR) may influence an individual’s perception of fairness to increase organizational trust. Previous studies have indicated the positive effect of GR (relative to NR) on trust. Future research can consider exploring the mediating role of trust in the relationship between non-balanced reciprocity and behavior. Furthermore, recent literature suggests that the social purpose of an organization plays an important role in connecting value ties between society and the organization (Thiel et al., 2021). Employees’ understanding of the organization’s social purpose (e.g., public service motivation; Thiel et al., 2021) may interfere in the formation of their compliance behavior. Self-interest (e.g., individual comparisons) and collective interest (e.g., social evaluation and social identity) may influence employees’ adherence to firm-wide rules (Thiel, 2021).

## Conclusion

The organization views non-balanced reciprocity as the pivotal exchange relationship norm for guiding employees to achieve beneficial behavior and desirable outcomes. Researching on non-balanced reciprocity (GR and NR) is more prevalent than on balanced reciprocity (Gervasi et al., 2022). Based on SET and CAT, we adopted an intrinsic self-regulatory approach and studied how and why non-balanced reciprocity influences employees’ compliance behavior. Our findings showed that GR (relative to NR) had positive effect on individuals’ compliance behavior and thriving at work. The results also proved that thriving at work mediated the relationship between GR and compliance behavior. We also took cognitive appraisal for technique and personality trait perspectives and investigated how the perceived cognitive capabilities of AI and conscientiousness moderated the impact of GR on thriving at work and the impact of thriving on compliance behavior.

## Data availability statement

The original contributions presented in the study are included in the article, and further inquiries can be directed to the corresponding author.

## Ethics statement

The studies involving human participants were reviewed and approved by Fujian Jiangxia University. The patients/participants provided their written informed consent to participate in this study.

## Author contributions

NZ contributed to development the theoretical model and data collection and analysis. YL, JZ, JiL, JuL, SW, and HG provided significant suggestions and improved the manuscript. All authors contributed to the article and approved the submitted version.

## Funding

The research was supported by Natural Science Foundation of Fujian Province (2022J01955), Educational and Scientific Research Project for Young and Middle-aged Teachers of Fujian Province (JAT210365), National Science Foundation Cultivation Program of Fujian Jiangxia University (JXS2021007), the National Natural Science Foundation of China (72074024), and the Key Project of National Social Science Foundation of China (22AZD026).

## Conflict of interest

The authors declare that the research was conducted in the absence of any commercial or financial relationships that could be construed as a potential conflict of interest.

## Publisher’s note

All claims expressed in this article are solely those of the authors and do not necessarily represent those of their affiliated organizations, or those of the publisher, the editors and the reviewers. Any product that may be evaluated in this article, or claim that may be made by its manufacturer, is not guaranteed or endorsed by the publisher.
